# Dimethyl-2-oxoglutarate improves redox balance and mitochondrial function in muscle pericytes of individuals with diabetes mellitus

**DOI:** 10.1007/s00125-020-05230-4

**Published:** 2020-07-30

**Authors:** Ashton Faulkner, Anita Tamiato, William Cathery, Andrea Rampin, Carlo Maria Caravaggi, Eva Jover, Steve Allen, Harry Mellor, David Hauton, Lisa C. Heather, Gaia Spinetti, Paolo Madeddu

**Affiliations:** 1 Bristol Medical School, Translational Health Sciences, University of Bristol, Upper Maudlin Street, Bristol, BS2 8HW UK; 2 School of Biochemistry, University of Bristol, University Walk, Bristol, BS8 1TD UK; 3 IRCCS, MultiMedica, Milan, Italy; 4 Department of Comparative Biomedical Sciences, Royal Veterinary College, London, UK; 5 Department of Chemistry, University of Oxford, Oxford, UK; 6 Department of Physiology, Anatomy & Genetics, University of Oxford, Oxford, UK

**Keywords:** 2-Oxoglutarate, Diabetes mellitus, Mitochondria, Pericytes, Redox, Vascular protection

## Abstract

**Aims/hypothesis:**

Treatment of vascular complications of diabetes remains inadequate. We reported that muscle pericytes (MPs) from limb muscles of vascular patients with diabetes mellitus display elevated levels of oxidative stress causing a dysfunctional phenotype. Here, we investigated whether treatment with dimethyl-2-oxoglutarate (DM-2OG), a tricarboxylic acid cycle metabolite with antioxidant properties, can restore a healthy metabolic and functional phenotype.

**Methods:**

MPs were isolated from limb muscles of diabetes patients with vascular disease (D-MPs) and from non-diabetic control participants (ND-MPs). Metabolic status was assessed in untreated and DM-2OG-treated (1 mmol/l) cells using an extracellular flux analyser and anion-exchange chromatography–mass spectrometry (IC-MS/MS). Redox status was measured using commercial kits and IC-MS/MS, with antioxidant and metabolic enzyme expression assessed by quantitative RT-PCR and western blotting. Myogenic differentiation and proliferation and pericyte–endothelial interaction were assessed as functional readouts.

**Results:**

D-MPs showed mitochondrial dysfunction, suppressed glycolytic activity and reduced reactive oxygen species-buffering capacity, but no suppression of antioxidant systems when compared with ND-MP controls. DM-2OG supplementation improved redox balance and mitochondrial function, without affecting glycolysis or antioxidant systems. Nonetheless, this was not enough for treated D-MPs to regain the level of proliferation and myogenic differentiation of ND-MPs. Interestingly, DM-2OG exerted a positive effect on pericyte–endothelial cell interaction in the co-culture angiogenesis assay, independent of the diabetic status.

**Conclusions/interpretation:**

These novel findings support the concept of using DM-2OG supplementation to improve pericyte redox balance and mitochondrial function, while concurrently allowing for enhanced pericyte–endothelial crosstalk. Such effects may help to prevent or slow down vasculopathy in skeletal muscles of people with diabetes.

**Figure:**
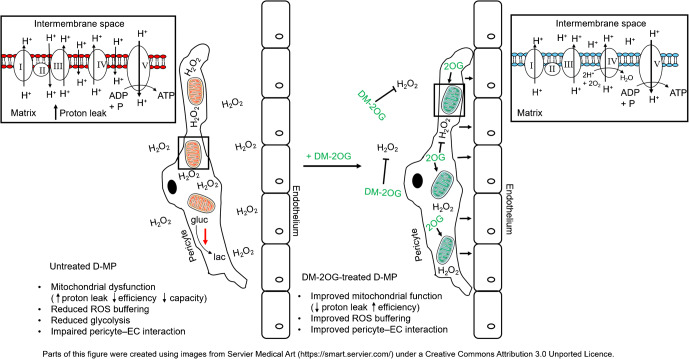
Graphical abstract

**Electronic supplementary material:**

The online version of this article (10.1007/s00125-020-05230-4) contains peer-reviewed but unedited supplementary material, which is available to authorised users.

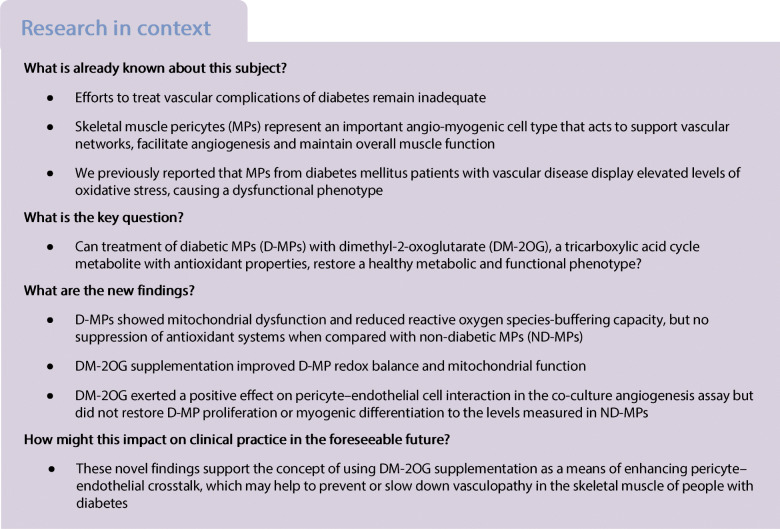



## Introduction

Globally, an estimated 463 million people are currently living with diabetes mellitus [1], a major risk factor for cardiovascular disease [2, 3]. This number is projected to rise over the next decade [1]. Muscle pericytes (MPs) represent an important angio-myogenic cell type that interact with endothelial cells (ECs) to support vascular networks and facilitate angiogenesis [4, 5]. Diabetes-driven disruption in pericyte–EC interaction is an important cause of vessel instability and ischaemia [6, 7]. The mechanisms involved are not fully understood but alterations in metabolic flexibility and induction of oxidative stress is thought to play a role [6, 8]. In line with this, we reported a reduction in number and function of supporting MP-like cells of diabetes patients presenting with critical limb ischaemia (CLI) [9]. Furthermore, these diabetic MPs (D-MPs) had elevated levels of reactive oxygen species (ROS) and exerted adverse effects on ECs.

Current treatments to reverse diabetic vascular complications remain sub-optimal, with approaches targeting metabolic and oxidative stress continuing to be actively pursued [8]. Given that the diabetic metabolic milieu can imprint on vascular cells, causing long-lasting changes beyond the restoration of systemic glucose control [10], a therapeutic option that sits at the metabolic/signalling interface may have significant impact. Metabolic intermediates of the tricarboxylic acid (TCA) cycle may represent one such interface. Among these, 2-oxoglutarate (2OG) may be a promising candidate.

In addition to its role in TCA cycle metabolism, 2OG has been shown to exert a protective effect through its ability to increase availability and activity of enzymatic and non-enzymatic antioxidant systems [11–14]. In this capacity, 2OG appears to have beneficial effects in models of ischaemic and oxidative stress [11, 13, 15–17]. Furthermore, supplementation with 2OG has been found to extend cellular lifespan by decreasing mitochondrial oxidative phosphorylation, indicating a possible beneficial effect on the ageing process [18]. More recently, 2OG has been suggested to improve the functional activity of cardiac mesenchymal stromal cells (MSCs) isolated from diabetic participants [19]. This effect was mediated, at least in part, through reducing the hyper-methylated state and improving glucose uptake and mitochondrial function, suggesting that this metabolite might prove beneficial as an additional therapy in diabetic individuals with cardiovascular disease. However, whether 2OG can exert beneficial effects on diabetes-induced pericyte dysfunction has not been investigated.

Given these perceived beneficial effects, the aim of this study was to characterise the metabolic and functional changes in pericyte-like cells isolated from diabetic participants presenting with CLI, and to investigate whether dimethyl-2OG (DM-2OG), a cell permeable 2OG derivative, could act as a ‘therapeutic metabolite’ to restore a healthy metabolic and functional phenotype.

## Methods

### Cell culture

Human samples were obtained with written informed consent. Their use conformed to the principles outlined in the Declaration of Helsinki and approved by the MultiMedica Research Ethics Committee (protocol #011/2009). MPs were isolated from: (1) different anatomical locations of the lower extremities from non-diabetic control participants, referred for investigations/therapeutic interventions related to leg varicosity; or (2) foot muscle from type 1 and type 2 diabetic participants at the occasion of minor amputation for CLI (diagnosed according to the Trans-Atlantic Inter-Society Consensus Document on Management of Peripheral Arterial Disease [TASC] 2007 guidelines). Characteristics of the study population can be found in electronic supplementary material (ESM) Table 1.

MP isolation was performed following a well-established procedure described previously [9, 20] (see ESM method: Muscle pericyte isolation). MPs were sub-cultured in α-minimum essential media (MEM) (20% vol./vol. FBS) and used for experimentation between passages 2 and 5. For all experiments, no randomisation protocol was applied and investigators were not blinded to the group assignment or to the experimental results.

HUVECs were purchased from Lonza (Slough, UK) and cultured on 1% gelatine in endothelial cell growth medium 2 (EGM2). Adult normal human dermal fibroblasts (NHDFs) were purchased from Promocell (Heidelberg, Germany) and expanded in fibroblast growth medium before being transferred to EGM2 for experimentation.

### PKCβII overexpression

HEK293-T cells expanded in DMEM (10% vol./vol. FBS) were transfected with a pLVX-puro plasmid (Clontech, UK) containing the human protein kinase C, isoform β II (PKCβII) sequence under the control of a cytomegalovirus (CMV) promoter. pLVX-puro-GFP was used as a control. Cells were collected 48 h post transfection and processed for RNA and protein analysis.

### Cell viability

MP viability was assessed using the Promega (Southampton, UK) LDH-glo Viability assay following the manufacturer’s instructions (see ESM method: Cell viability).

### Functional analysis

#### Angiogenesis

Angiogenesis was assessed using the 3D co-culture angiogenesis assay as previously described [21, 22]. NHDFs were cultured to confluence in EGM2 in 24-well plates. MPs and HUVECs (1:4 ratio) were seeded in EGM2 directly on top of the confluent NHDFs (technical duplicate). MPs were pre-stained with DiI (1,1′-dioctadecyl-3,3,3′,3′-tetramethylindocarbocyanine perchlorate; 1:200; 20 min). Following incubation for 7 days (medium and treatment refreshed every 48 h), cells were fixed using 4% (wt/vol.) paraformaldehyde (PFA), and vessel structures were viewed by staining with anti-platelet endothelial cell adhesion molecule (PECAM) antibody (R&D systems BBA7; 1:200). Images were acquired using a Zeiss Axio Observer.Z1 fluorescence microscope (Carl Zeiss, UK) and analysed manually using ImageJ (v 1.52) software (https://imagej.nih.gov/ij/index.html).

#### Proliferation

MPs were seeded in 96-well plates (1000 cells/well in triplicate) in the absence or presence of 1 mmol/l DM-2OG (Sigma Aldrich, UK). Proliferation over 72 h was quantified using a BrdU incorporation kit (Sigma Aldrich) following the manufacturer’s instructions. Medium and treatment were refreshed every 24 h. BrdU (10 μmol/l) was added for the final 24 h (see ESM method: Proliferation).

#### Myogenic differentiation

MPs were seeded in 8-well chamber slides and cultured at confluence for 7 days to allow cell fusion and myotube formation. Cells were fixed with 4% (wt/vol.) PFA and permeabilised with 0.3% (vol./vol.) Triton X-100. Myogenic differentiation was assessed by probing cells overnight (4°C) with anti-myosin heavy chain (MyHC) antibody (R&D Systems MAB4470; clone MF20; 1:200). Nuclei were counter-stained using DAPI. Images were acquired using a Zeiss Axio Observer.Z1 fluorescence microscope and analysed using ImageJ software.

#### Permeability

Permeability was assessed by measuring the transfer of FITC-dextran (4 kDa or 70 kDa) through HUVEC monolayers in transwell tissue culture plates (see ESM method: Permeability).

### RT-qPCR

RNA was extracted using the Qiagen RNeasy Plus Mini Kit (Qiagen, UK) according to the manufacturer’s instructions. Complementary DNA was synthesised using SuperScript II Reverse Transcriptase (ThermoFisher Scientific, UK). Quantitative RT-PCR (RT-qPCR), using intercalator (SYBR Premix Ex-Taq; TaKara Bio, France) and TaqMan technologies, was performed to determine changes in gene expression. *PRKCB* (Hs00176998_m1) and *TBP* (Hs00427620_m1) TaqMan primer-probes were obtained from ThermoFisher Scientific and *SHC1* (H1_SHC1) was obtained from Sigma Aldrich. All other primer sequences were designed using Primer3 version 4.1.0 (http://primer3.ut.ee/) and NCBI primer-BLAST software (https://www.ncbi.nlm.nih.gov/tools/primer-blast/) or obtained from published literature (ESM Table 2). Analysis was performed using the ${2}^{-{\Delta \Delta \mathrm{C}}_t}$ method with results normalised to *TBP* control gene.

### Western blotting

Cells were lysed in RIPA buffer supplemented with protease/phosphatase inhibitor cocktail (Sigma Aldrich). Protein concentration was determined by bicinchoninic acid (BCA) Protein Assay (ThermoFisher Scientific). Proteins (25 μg) were separated by SDS-PAGE (60–90 min; 120 V). Resolved proteins were transferred to PVDF membranes (0.22 μm; GE Healthcare, UK) by semi-dry transfer (90 min; 22 V). Membranes were blocked (5% wt/vol. skimmed (non-fat) milk; 1 h) and probed with primary antibodies overnight (4°C) (ESM Table 3) followed by incubation with ECL-HRP-conjugated secondary antibodies (1:10,000; 1 h) (GE Healthcare). Proteins were detected by enhanced chemiluminescence using Amersham ECL reagent (GE Healthcare). Band densities were semi-quantified by densitometry using ImageJ and normalised to β-actin loading control.

### Immunocytochemistry

Cells were fixed in 4% (wt/vol.) PFA (10–15 min) and permeabilised in 0.1% (vol./vol.) Triton X-100 (15 min). Cells were incubated in blocking buffer (5% vol./vol. FBS in PBS-Tween20; 45 min) prior to overnight (4°C) incubation with primary antibody at optimised concentrations (ESM Table 4). Primary antibodies were detected with Alexa-Fluor-488, -568 or -647 secondary antibodies (1:400 in PBS-Tween20) (ThermoFisher Scientific). Samples were incubated with DAPI (1:1000; 3 min) and images were collected using a Zeiss Axio Observer.Z1 fluorescence microscope.

### Metabolic analysis

#### Anion-exchange chromatography–mass-spectrometry

Metabolites were measured using anion-exchange chromatography–mass-spectrometry (IC-MS/MS), similar to that described previously [23]. Compounds were identified with reference to accurate mass and retention time compared with authenticated standards run in-house. All data were normalised to total DNA for each sample and expressed as relative abundance. Absolute abundance of adenine nucleotides (ATP/AMP/ADP) was calculated with reference to standard curves (see ESM method: Anion-exchange chromatography Mass-Spectroscopy).

#### Seahorse extracellular flux analysis

Changes in oxygen consumption rate (OCR) and extracellular acidification rate (ECAR) were measured using the Seahorse XFp extracellular flux analyzer (Agilent, UK). Baseline readings were obtained, followed by sequential addition of oligomycin (2 μmol/l), carbonyl cyanide-4-(trifluoromethoxy)phenylhydrazone (FCCP; 2 μmol/l) and rotenone/antimycin-A (0.5 μmol/l each). Three readings (5 min intervals) were obtained after each pharmacological agent and values normalised to protein content (see ESM method: Seahorse extracellular flux analysis).

#### Extracellular lactate accumulation

Medium lactate was measured using ABX-Pentra-C200 chemistry analyser (HORIBA, UK) with results normalised to protein content (see ESM method: Extracellular lactate accumulation).

#### Measurement of H_2_O_2_

Cells (1000 cells/well 96-well plate) were incubated in the presence or absence of DM-2OG for 16 h (in triplicate). Medium H_2_O_2_ level was measured using the Promega ROS-Glo H_2_O_2_ Assay following the manufacturer’s instructions (see ESM method: Measurement of H_2_O_2_).

#### Measurement of GSH/GSSG

Cells were incubated for 16 h in the absence or presence of 1 mmol/l DM-2OG. Glutathione (GSH) was measured using the Promega GSH/GSSG-Glo Assay kit following the manufacturer’s instructions. Results were normalised to protein content and the reduced GSH/oxidised GSH (GSSG) ratio was calculated as GSH-GSSG/(GSSG/2) (see ESM method: Measurement of GSH/GSSG).

### Mitochondrial assessment

#### Mitochondrial DNA content

Following extraction of DNA (QIAamp DNA Mini Kit; Qiagen, UK), a measure of mitochondrial to genomic DNA was performed by RT-qPCR using primers directed against mitochondrial-encoded tRNA-Leu(UUR) and nuclear-encoded B2-microglobulin, as described by Rooney et al [24].

#### Mitochondrial visualisation

The mitochondrial-specific stain, MitoTracker Red-CMXRos (ThermoFisher Scientific), was used to visualise gross mitochondrial morphology by fluorescence microscopy. Cells were incubated in α-MEM containing 50 nmol/l MitoTracker Red-CMXRos dye (30 min). Images were acquired using a Zeiss Axio Observer.Z1 fluorescence microscope (×40 objective).

### Statistical analysis

Data are expressed as means ± SEM from independent experiments performed on cell isolates from separate donors. Data analysis was performed using GraphPad Prism 6 software. Student’s *t* test was employed for establishing significant differences between two groups. For comparison of three or more groups, ANOVA was performed followed by an appropriate post hoc test. In all cases, *p* < 0.05 indicated statistical significance.

## Results

### Isolated cells display typical pericyte-related markers

MP-like cells isolated from diabetic patients presenting with CLI (D-MPs), together with non-diabetic control participants (ND-MPs), were screened for a combination of pericyte-associated markers. In line with our previous report [9], isolated cells were confirmed to be positive for platelet-derived growth factor receptor β (PDGFRβ), neuron-glial antigen (NG2) and CD146, and negative for endothelial marker CD31 (ESM Fig. 1). Expression of these markers did not differ between ND- and D-MPs (ESM Fig. 2a). Importantly, viability was similar between ND- and D-MP and was not affected by 1 mmol/l DM-2OG (ESM Fig. 2b), a concentration comparable with that of other studies [25–27].

### Mitochondrial function in D-MPs is improved by DM-2OG

**Fig. 1:**
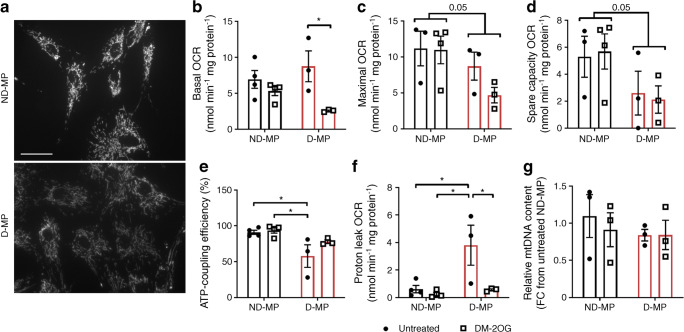
(**a**) Representative images of MitoTracker-stained cells showing that gross mitochondrial morphology is not different between ND- and D-MPs; scale bar, 20 μm. (**b**) Basal OCR is not different between untreated ND- and D-MPs but is significantly reduced in D-MPs supplemented with DM-2OG (1 mmol/l; 16 h). (**c**) Maximal OCR and (**d**) spare capacity OCR are lower in D-MPs with no effect of DM-2OG supplementation. (**e**) ATP-coupling efficiency is significantly lower in D-MPs and is partially improved following DM-2OG supplementation. (**f**) Mitochondrial proton leak is significantly higher in D-MPs and is restored towards the level of ND-MPs following supplementation with DM-2OG. (**g**) No change in relative mitochondrial DNA content was observed between ND- and D-MPs with or without DM-2OG treatment. Data are means (± SEM) of *n* = 3–4 for ND-MP and *n* = 3 for D-MP; *p < 0.05 as determined by two-way ANOVA followed by Tukey’s post-comparison test. Numbers above bars in (**c**, **d**) indicate *p* values. FC, fold change

Mitochondrial network morphology was similar between ND- and D-MPs (Fig. 1a), and no significant difference was observed in basal mitochondrial OCR (Fig. 1b). However, maximal OCR and spare capacity were reduced in D-MPs compared with ND-MPs (Fig. 1c, d). OCR coupled to ATP production was variable in D-MPs but when considered as a percentage of basal OCR (ATP-coupling efficiency), was significantly reduced compared with ND-MPs (Fig. 1e). Furthermore, D-MPs also showed a significant increase in non-ATP-linked OCR (mitochondrial proton leak) (Fig. 1f). Following supplementation with DM-2OG (16 h), basal OCR was reduced in D-MPs while maximal and spare capacity were largely unaffected. Moreover, the level of proton leak was significantly reduced back towards that measured in ND-MPs. These changes were associated with a partial improvement in ATP-coupling efficiency, although ATP, ADP and AMP levels did not differ between ND- and D-MPs or following supplementation with DM-2OG (ESM Fig. 3). In addition, no effect of DM-2OG treatment was observed in ND-MPs (Fig. 1). Importantly, mitochondrial DNA content was similar between all treatment conditions (Fig. 1g), suggesting mitochondrial content remained unchanged.

**Fig. 2:**
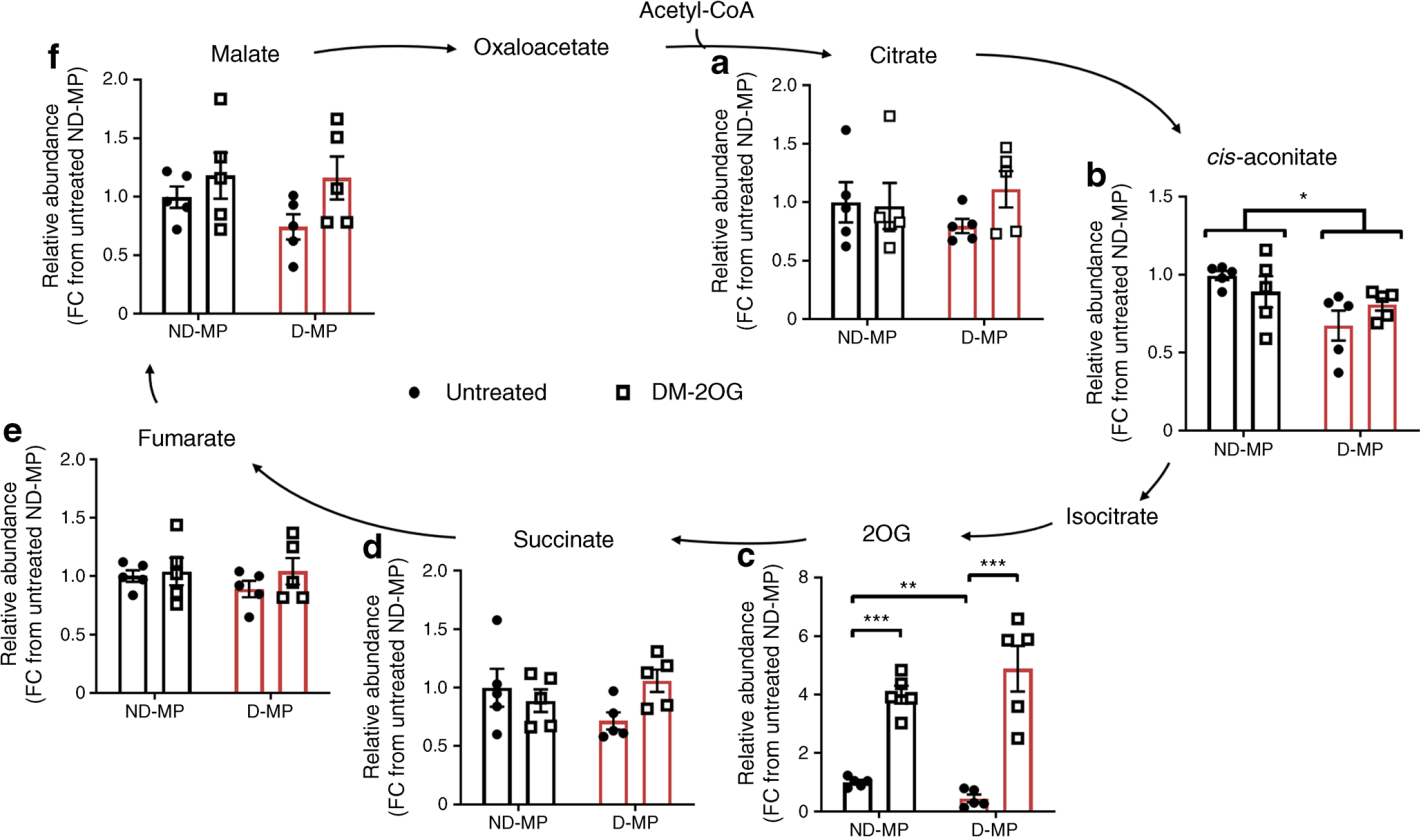
ND- and D-MPs have similar levels of citrate (**a**), succinate (**d**), fumarate (**e**) and malate (**f**). D-MPs have significantly reduced levels of endogenous *cis*-aconitate (**b**) and 2OG (**c**) compared with ND-MPs. Supplementation with exogenous DM-2OG (1 mmol/l; 16 h) significantly increased the intracellular abundance of 2OG in both ND- and D-MPs without affecting other TCA cycle intermediates. Data represent means (± SEM) of *n* = 5; *p < 0.05, **p < 0.01, ***p < 0.001 as determined by two-way ANOVA followed by Student’s *t* test. FC, fold change

To understand if changes in OCR were linked with alterations in TCA cycle activity, metabolite abundance was measured by IC-MS/MS. No difference was found in the level of citrate, succinate, fumarate or malate (Fig. 2). However, abundance of *cis*-aconitate and 2OG were significantly reduced in D-MPs. Following supplementation with exogenous DM-2OG, intracellular abundance of 2OG was significantly increased in both ND- and D-MPs, with no change in any other metabolite.

### Altered mitochondrial function is not a consequence of PKCβII/p66^SHC^ activation

Upregulation of the PKCβII/p66^SHC^ pathway has been reported as a prominent modulator of mitochondrial function and ROS production [28–30]. However, RT-qPCR and western blot analysis revealed that MPs did not express PKCβII to an extent that could be reliably detected (ESM Fig. 4). Adequate performance of primers and antibody against human PKCβII was confirmed using an overexpression system in HEK293-T cells. Furthermore, a significant reduction in the expression of the PKCβII target, p66^SHC^, was seen in D-MPs (ESM Fig. 4). These data suggest that this pathway is unlikely to be a significant contributing factor to the altered mitochondrial function observed in the D-MPs.

### Impaired glycolysis and metabolic flexibility in D-MPs is not rescued by DM-2OG

As glycolysis works alongside mitochondria to sustain vascular cell function [31, 32], and diabetes is known to affect this reciprocal interaction [33], we next investigated the impact of diabetes on markers of glycolysis and the effect of DM-2OG supplementation.

**Fig. 3:**
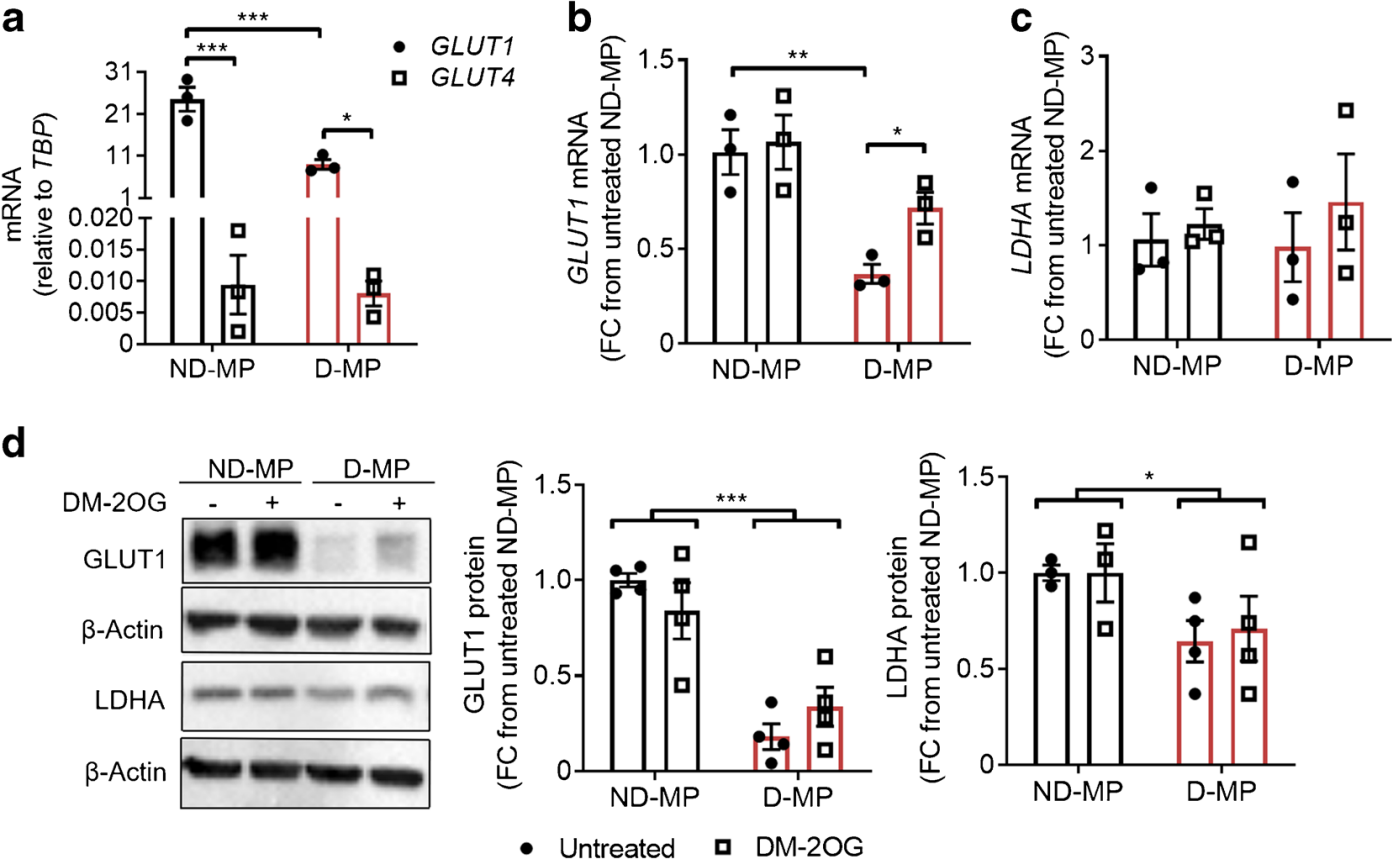
(**a**) ND- and D-MPs have greater levels of *GLUT1* compared with *GLUT4* at the mRNA level. (**b**) D-MPs have a significantly reduced level of *GLUT1* mRNA expression compared with ND-MPs. Supplementation with DM-2OG (1 mmol/l; 16 h) significantly increases *GLUT1* mRNA expression in D-MPs with no effect on ND-MPs. (**c**) *LDHA* mRNA expression is not significantly different between ND- and D-MPs with or without DM-20G supplementation. (**d**) Representative western blots showing GLUT1, LDHA and β-actin loading control (see ESM Fig. 7 for original unedited blots) and associated densitometry analysis showing that D-MPs have a significantly reduced expression of GLUT1 and LDHA protein compared with ND-MPs, with no significant effect of DM-2OG supplementation. Data are means (± SEM) of *n* = 3–4; *p < 0.05, **p < 0.01, ***p < 0.001 as determined by two-way ANOVA followed by Bonferroni’s post-comparison test. FC, fold change

**Fig. 4:**
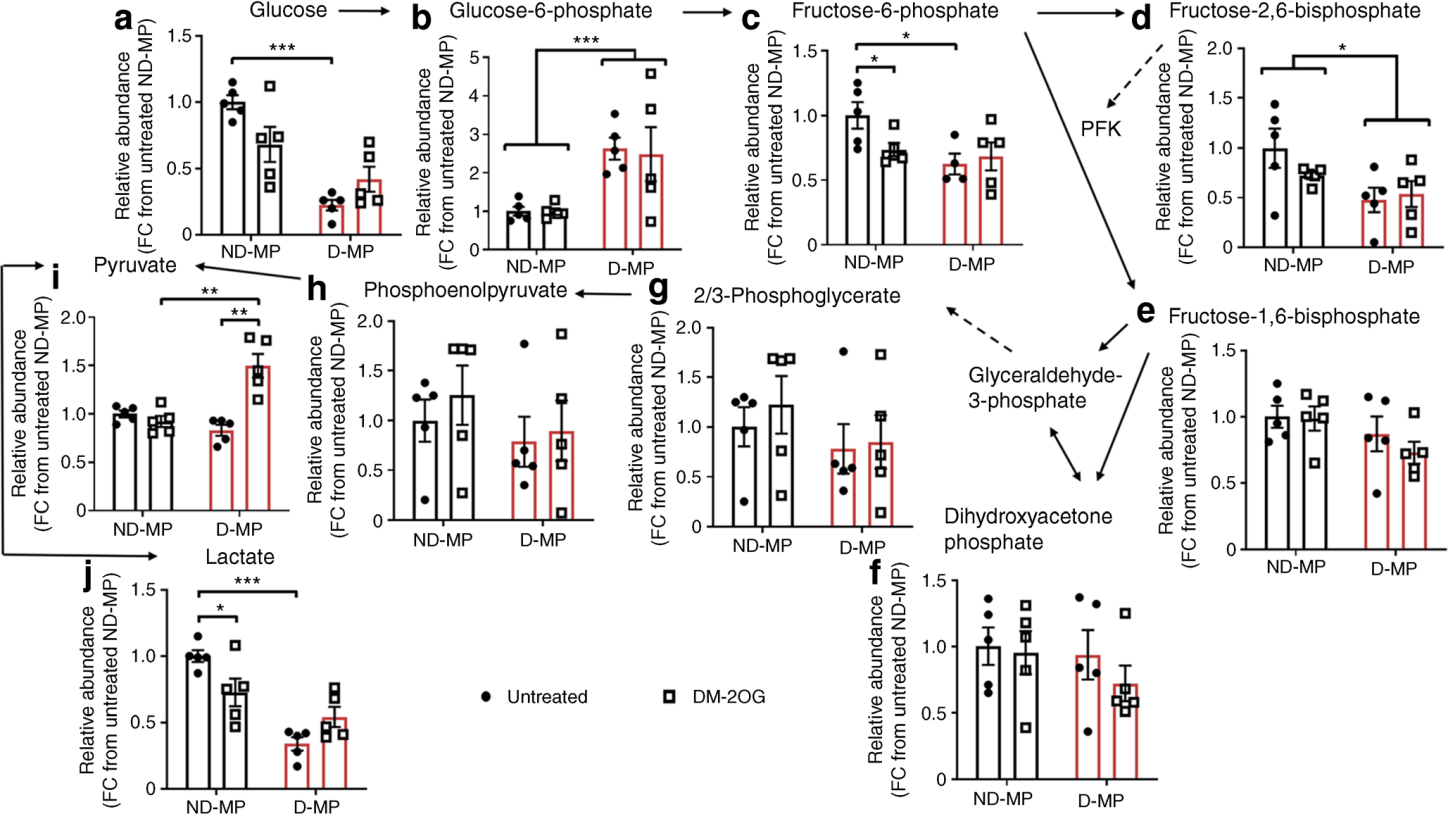
Relative abundance of some glycolysis intermediates (**a**–**j**) is significantly altered in D-MPs compared with ND-MPs, indicating reduced glycolytic activity. DM-2OG supplementation (1 mmol/l; 16 h) significantly reduces the abundance of fructose-6-phosphate (**c**) and lactate (**j**) in ND-MPs but increases pyruvate abundance (**i**) in D-MPs. Data represent means (± SEM) of *n* = 5; *p < 0.05, **p < 0.01, ***p < 0.001 as determined by two-way ANOVA followed by Student’s *t* test. FC, fold change; PFK, phosphofructokinase

MPs expressed greater levels of the *GLUT1* transporter compared with *GLUT4* (Fig. 3a), and D-MPs had significantly less GLUT1 compared with ND-MPs at both mRNA and protein levels (Fig. 3b, d). D-MPs also had reduced lactate dehydrogenase A (LDHA) protein expression, but not mRNA, compared with ND-MPs (Fig. 3c, d). Following supplementation with DM-2OG (16 h), *GLUT1* mRNA was restored in D-MPs, however, this was not observed at the protein level. Moreover, no effect of DM-2OG was observed on LDHA expression in D-MPs or on GLUT1 and LDHA expression in ND-MPs (Fig. 3b–d). Analysis of glycolytic intermediates revealed a significant reduction in glucose, fructose-6-phosphate, fructose-2,6-bisphosphate and lactate in D-MPs, and an accumulation in glucose 6-phosphate, further indicating supressed glycolytic activity (Fig. 4). Following supplementation with DM-2OG, ND-MPs had reduced abundance of fructose-6-phosphate and lactate, suggesting elevated 2OG level reduces glycolytic activity (Fig. 4). In contrast, DM-2OG-treated D-MPs showed no alteration in metabolite abundance, except for an elevation in pyruvate, which may be the result of altered mitochondrial metabolism or change in other metabolic pathways involving this metabolite.

To understand if glycolytic capacity and metabolic flexibility were impacted, the ability to upregulate anaerobic glycolysis was investigated by measuring the change in lactate release under hypoxia (2% O_2_). Compared with ND-MPs, D-MPs had reduced lactate release under normoxia and hypoxia, despite both significantly increasing mRNA expression of the hypoxia-inducible factor 1 α (HIF1α) target, *GLUT1* (ESM Fig. 5a–c). Furthermore, while DM-2OG had no effect on lactate release by D-MPs, it was significantly reduced in ND-MPs under normoxic and hypoxic conditions (ESM Fig. 5a). However, the degree of upregulation following hypoxia was similar between untreated and DM-2OG-treated ND-MPs (ESM Fig. 5d). A similar change was observed when monitoring ECAR, a surrogate readout of lactate release, following pharmacological inhibition (oligomycin; 2 μmol/l) of ATP-synthase activity (ESM Fig. 5e).

These data suggest that D-MPs have lower glycolytic activity and reduced metabolic flexibility, with less capacity for upregulating glycolysis when required. Supplementation with DM-2OG has little effect on D-MPs but lowers glycolytic activity in ND-MPs, without affecting their ability to respond to hypoxia.

### DM-2OG has beneficial effects on D-MP redox status without upregulating antioxidant systems

We previously demonstrated increased ROS burden in D-MPs [9], and given the changes in metabolic status described above, we further investigated the redox status in D-MPs and the ability of DM-2OG to restore redox balance.

**Fig. 5:**
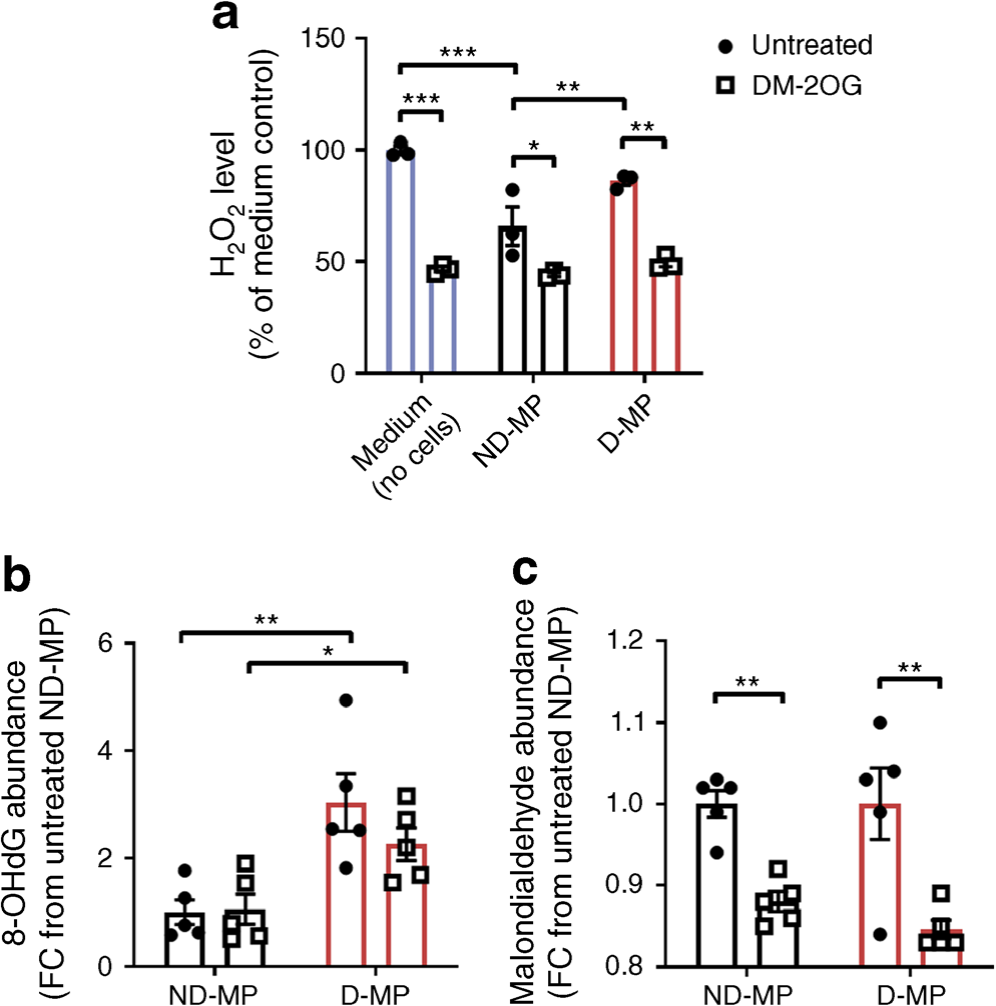
(**a**) D-MPs have reduced H_2_O_2_ buffering activity compared with ND-MPs. Supplementation with DM-2OG (1 mmol/l; 16 h) significantly reduces medium H_2_O_2_ level under all experimental conditions. Data represent mean (± SEM) percentage of H_2_O_2_ level from untreated medium control; *n* = 3; *p < 0.05, **p < 0.01, ***p < 0.001 as determined by two-way ANOVA followed by Bonferroni’s post-comparison test. (**b**) D-MPs have significantly greater levels of 8-OHdG, a marker of oxidative DNA damage, with levels unaffected by DM-2OG supplementation. (**c**) DM-2OG supplementation significantly reduces the relative abundance of malondialdehyde, a marker of lipid peroxidation, in both ND- and D-MPs. For (**b**, **c**) data represent means (± SEM) of *n* = 5; *p < 0.05, **p < 0.01 as determined by two-way ANOVA followed by Student’s *t* test. FC, fold change

D-MPs displayed reduced ability to buffer H_2_O_2_ within the culture medium compared with ND-MPs (Fig. 5a) and contained higher levels of 8-hydroxy-2′-deoxyguanosine (8-OHdG), a marker of oxidative DNA damage (Fig. 5b). No difference was seen in the level of malondialdehyde, an indicator of lipid peroxidation (Fig. 5c). Supplementation with DM-2OG significantly reduced medium H_2_O_2_ under all experimental conditions (Fig. 5a). However, DM-2OG did not affect 8-OHdG level but significantly reduced malondialdehyde in both ND- and D-MPs (Fig. 5b, c).

**Fig. 6:**
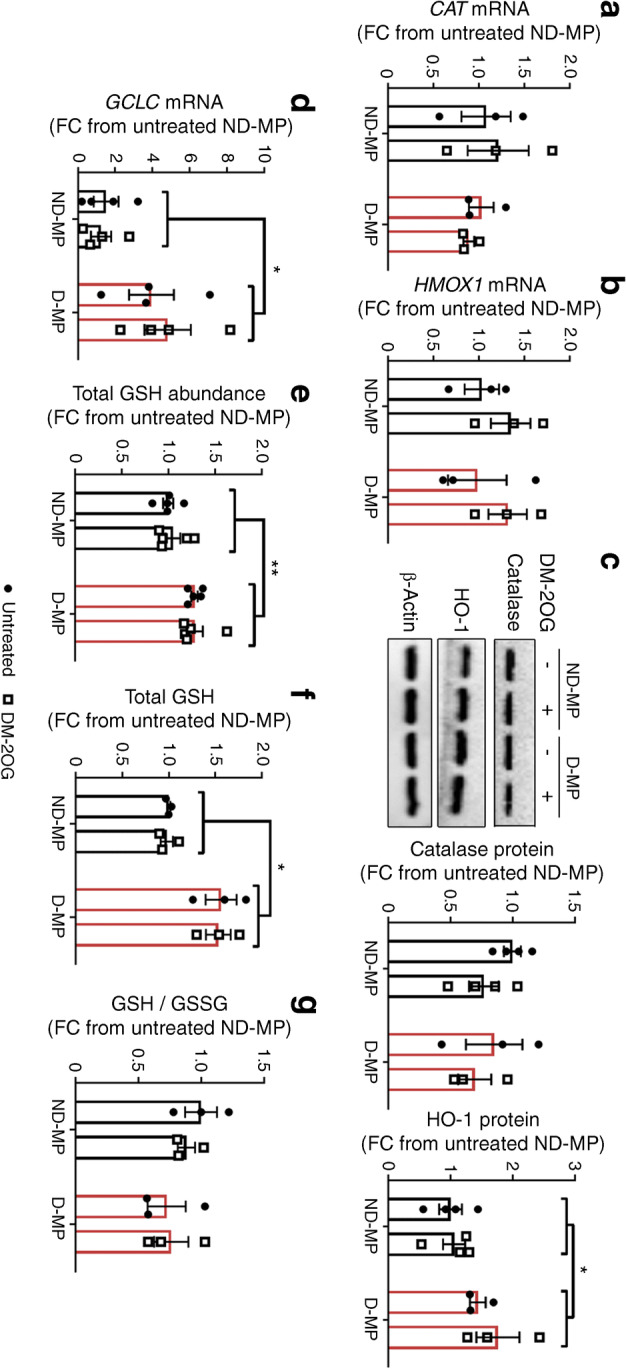
(**a**) *CAT* and (**b**) *HMOX1* mRNA expression do not differ between ND- and D-MPs or following supplementation with DM-2OG (1 mmol/l; 16 h). Data represent means (± SEM) from *n* = 3. (**c**) Representative western blot and associated densitometry analysis showing that HO-1 protein, but not catalase, is significantly increased in D-MPs with no effect of DM-2OG supplementation. Data represent means (± SEM) from *n* = 4 for ND-MP and *n* = 3 for D-MP; *p < 0.05 as determined by two-way ANOVA followed by Bonferroni’s post-comparison test. See ESM Fig. 7 for original unedited blots. (**d**) *GCLC* mRNA expression is significantly higher in D-MPs, with no effect of DM-2OG supplementation. Data represent means (± SEM) from *n* = 4; *p < 0.05 as determined by two-way ANOVA followed by Bonferroni’s post-comparison test. (**e**) IC-MS/MS (*n* = 5) and (**f**) GSH-Glo assay (*n* = 3) analysis showing that total GSH is significantly higher in D-MPs, with no effect of DM-2OG supplementation. (**g**) The GSH/GSSG ratio (*n* = 3) is not significantly different between ND- and D-MPs or following supplementation with DM-2OG. For (**e**–**g**) data represent means (± SEM); *p < 0.05, **p < 0.01 as determined by two-way ANOVA. FC, fold change

To identify if the effects of DM-2OG were due to upregulating antioxidant defence, we assessed MP antioxidant status. ND- and D-MPs had similar amounts of the H_2_O_2_-scavenging enzyme, catalase, at both the mRNA and protein level (Fig. 6a, c). In contrast, D-MPs had a small, but significant, increase in haemoxygenase-1 (HO-1) protein, a central antioxidant and cyto-protective enzyme, while mRNA levels remained unchanged (Fig. 6b, c). Furthermore, mRNA expression of the catalytic unit of a rate-governing enzyme of glutathione synthesis, glutamate-cysteine ligase (*GCLC*) (Fig. 6d), together with total GSH levels, as measured by IC-MS/MS (Fig. 6e) and the GSH/GSSG-Glo assay (Fig. 6f), were significantly increased in D-MPs compared with ND-MPs. Despite this upregulation, the reduced to oxidised GSH ratio (GSH/GSSG) was not significantly different (Fig. 6g). Importantly, none of these parameters were significantly altered by DM-2OG, suggesting that the beneficial effect of DM-2OG on ROS status is unlikely to be due to inducing antioxidant defence systems.

### Pericyte–endothelial interaction is enhanced by DM-2OG

To understand whether the changes in metabolic and redox homeostasis are associated with altered functional activity, in vitro assessment of proliferation, myogenic differentiation and pericyte–endothelial interaction was performed.

**Fig. 7:**
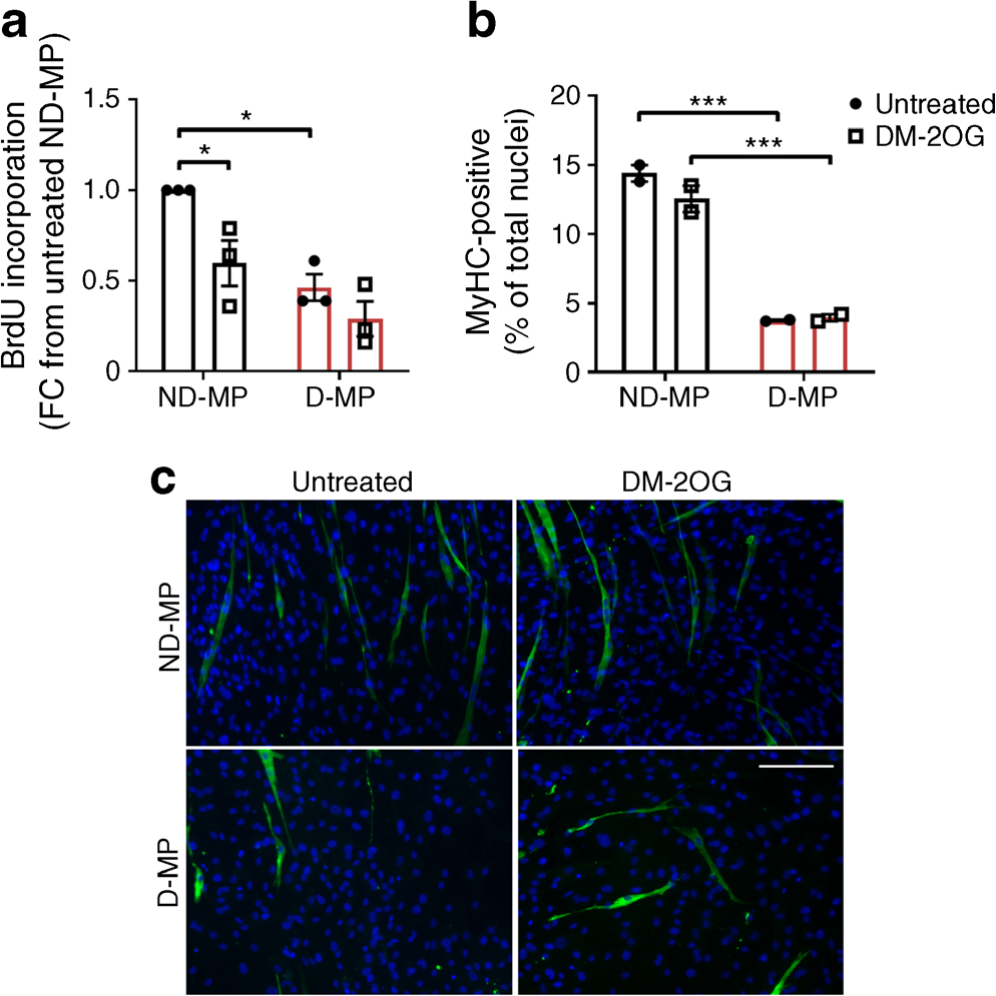
(**a**) D-MPs show significantly reduced proliferation over 72 h compared with ND-MPs. Supplementation with DM-2OG (1 mmol/l; 72 h) significantly reduces proliferation of ND- but not D-MPs. Data represent means (± SEM) fold-change in BrdU incorporation from *n* = 3; *p < 0.05 as determined by two-way ANOVA followed by Tukey’s post-comparison test. (**b**) Analysis and (**c**) representative immunofluorescence images (scale bar, 50 μm) showing that D-MPs have reduced propensity to differentiate down the myogenic lineage compared with ND-MPs at 7 days, with no effect of DM-2OG supplementation. Data represent means (± SEM) percentage of nuclei associated with positive MyHC staining (green) from *n* = 2; ***p < 0.001 as determined by two-way ANOVA followed by Bonferroni’s post-comparison test. FC, fold change

D-MPs displayed a significantly reduced rate of proliferation compared with ND-MPs, and supplementation with DM-2OG had a significant inhibitory effect on ND- but not D-MPs (Fig. 7a). Similarly, D-MPs showed less propensity to differentiate down the skeletal myocyte lineage, with no effect of DM-2OG being observed (Fig. 7b, c).

**Fig. 8:**
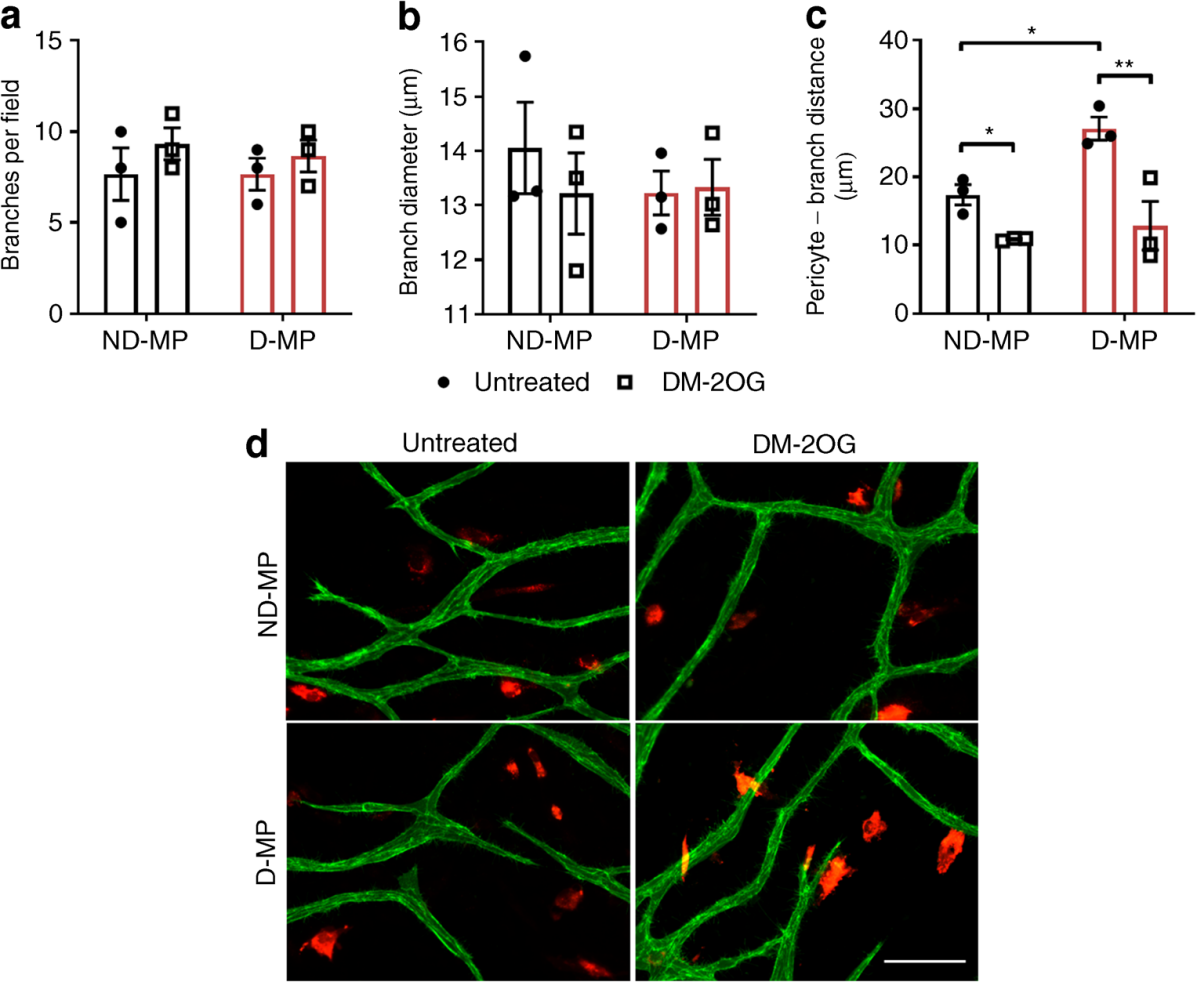
No significant difference was observed between wells containing ND- or D-MPs with regard to the mean number of branches (**a**) or mean branch diameter (**b**) formed by HUVECs in the 3D angiogenesis assay. (**c**) Quantification and (**d**) representative immunofluorescence images (scale bar, 50 μm) showing that the mean distance between pericytes (red; DiI) and HUVEC branches (green; PECAM) is significantly greater in wells containing D-MPs compared with ND-MPs and is significantly reduced when supplemented with DM-2OG (1 mmol/l; 7 days). Data represent means (± SEM) from *n* = 3; *p < 0.05, **p < 0.01 as determined by two-way ANOVA followed by Bonferroni’s post-comparison test

Given our previous identification of an adverse impact of D-MPs on HUVEC 2D network formation [9], we further assessed the impact of MPs on EC angiogenic behaviour in a 3D co-culture assay. The mean number and diameter of capillary-like tubes was not significantly different between wells containing ND- or D-MPs or following supplementation with DM-2OG (Fig. 8a, b). However, although MPs showed evidence of direct interaction with ECs, the mean distance between the MP and endothelial network was significantly greater with D-MPs (Fig. 8c, d), indicating reduced interaction/communication. This distance was significantly reduced in the presence of DM-2OG, an effect seen with both D- and ND-MPs, suggesting that DM-2OG can improve pericyte–endothelial interaction/crosstalk.

To assess whether improved interaction influences endothelial barrier properties, the effect of MPs and DM-2OG on HUVEC permeability was investigated. The presence of MPs significantly reduced the ability of large molecules (represented by 70 kDa FITC-dextran) to pass through a HUVEC monolayer but had no effect on smaller molecules (represented by 4 kDa FITC-dextran) (ESM Fig. 6). Moreover, no significant difference was seen between wells containing ND- or D-MPs, or those containing DM-2OG.

Together these data demonstrate that D-MPs have reduced proliferation and differentiation capacity and that DM-2OG supplementation is not able to rescue these deficiencies. D-MPs do not adversely impact permeability or the angiogenic behaviour of healthy ECs but do display less interaction with the endothelial network, a deficit that is significantly improved by DM-2OG.

## Discussion

The aim of the present study was to characterise the metabolic and functional changes in D-MPs and identify any beneficial effect of DM-2OG supplementation.

While the presence of D-MPs did not alter EC permeability or the ability of ECs to undergo tubulogenesis, D-MPs did demonstrate a reduction in their proliferative behaviour, reduced propensity to differentiate down the myogenic lineage, and reduced interaction with ECs. This impairment in function is not due to any significant change in cell viability, but instead can be explained, at least in part, through the observed changes in D-MP metabolic phenotype imparted by the diabetic milieu and probably compounded by the ischaemic environment from which the cells are isolated.

Cells utilise mitochondria alongside glycolysis to facilitate signalling and anabolic metabolism aimed at driving proliferation, migration and morphological re-arrangement [31, 32, 34]. We report that D-MPs have significant alterations in both glycolysis and mitochondrial metabolism that are in line with the observed decline in cell function. In agreement with earlier studies [35, 36], MPs predominantly expressed the GLUT1 transporter that, alongside LDHA, was significantly downregulated in D-MPs. Alongside this, D-MPs displayed reduced glycolytic activity and a lack of metabolic flexibility. Furthermore, D-MPs also presented with significant changes in mitochondrial function. Although mitochondrial network morphology and basal OCR was not significantly compromised, D-MPs had reduced maximal and spare OCR capacities, together with reduced efficiency of oxygen-linked ATP production. D-MPs also showed an elevation in proton leak, an additional indicator of oxidative burden [37, 38]. Such blunted metabolic capacity would contribute to the reduced functional activity of D-MPs. Indeed, the importance of upregulating these metabolic networks for such behaviour has been demonstrated in other vascular cells [31, 32, 34].

Although it was not possible to identify a specific inducer of the mitochondrial dysfunction, it is possible to rule out activation of PKCβII, as we were unable to detect this protein kinase. Unlike other cells, the role of the PKCβII/p66^SHC^ pathway in pericytes is less studied and mainly restricted to the retina [39–41]. Even when detected, its role in pericyte biology and diabetes-related dysfunction remains unclear [41, 42].

Alterations in metabolism are often accompanied by changes in redox status/control that have a profound impact on vascular function [43]. We have shown that D-MPs have higher levels of oxidative stress [9], and in the present study, D-MPs displayed a greater level of oxidative damage, particularly towards DNA, and reduced capacity to buffer their environment. Importantly, this reduction in ROS-buffering does not appear to be due to compromised expression of antioxidant systems.

Given the potential role of 2OG as an antioxidant and signalling molecule [11–14, 44], we explored whether DM-2OG supplementation could exert a beneficial effect on metabolic balance to allow for restoration of D-MP function. This is particularly pertinent as endogenous 2OG abundance was reduced in D-MPs, suggesting that a lack of this metabolite may be a compounding factor in the observed functional decline.

Following DM-2OG supplementation, intracellular abundance of 2OG was elevated. However, the lack of an effect on antioxidant systems contrasts with reports in other cell types [12, 14]. Instead, it is possible that DM-2OG is acting, at least in part, as a direct scavenger of H_2_O_2_, as indicated by the reduction in H_2_O_2_ levels within the culture medium. Indeed, by virtue of its structure, direct 2OG interaction and clearance of H_2_O_2_ has previously been identified, presenting a complementary antioxidant mechanism [25, 45].

Reduction in oxidative stress is also consistent with the reduction in mitochondrial OCR and improved efficiency of oxygen-coupled ATP production observed in DM-2OG-treated D-MPs, as lower OCR and more efficient mitochondria are less likely to produce excessive ROS [46]. A similar effect on oxygen utilisation has been reported previously, where interaction between 2OG and mitochondrial ATP-synthase was described [18]. In the current study, DM-2OG supplementation also led to reduced OCR linked to proton leak, indicating reduced oxidative burden and improved mitochondrial function. The level of malondialdehyde was also lowered following DM-2OG supplementation, suggesting a potential protective effect on lipid peroxidation state, which could also benefit mitochondria. It is worth noting that apart from the reduction in malondialdehyde and medium H_2_O_2_ level, none of the above effects were evident in ND-MPs. This was not due to reduced DM-2OG uptake but is likely to be a consequence of its differential utilisation, with greater effects being imparted on reducing glucose metabolism. Although the reduction in glycolysis could be through 2OG acting as a positive co-factor for prolyl hydroxylase-mediated degradation of HIF1α, this does not appear to prevent the ability to respond to hypoxia or upregulate *GLUT1* expression (a HIF1α target) when required.

Given these differential metabolic effects, it is not surprising that DM-2OG was unable to restore D-MP proliferation and reduced proliferation of ND-MPs. The effect of 2OG on cell proliferation is variable within the literature [47–49], and our results contrast with those reported in cardiac MSCs, where 2OG was found to have no effect on metabolic or proliferative activity of non-diabetic MSCs, but rescued proliferation of diabetic MSCs in association with a rescue of glucose metabolism and mitochondrial function [19].

Importantly, DM-2OG was able to enhance pericyte–EC interaction in the angiogenesis assay. Although the mechanism needs identifying through further investigation, it is likely to involve a combination of effects imparted on both MPs and ECs. Interestingly, while investigating 2OG for preventing tumour progression in mice, greater pericyte coverage of vessels was observed following 2OG treatment [50]. When coupled with our own observation, this may indicate a potential role for 2OG in facilitating pericyte–endothelial interaction and vessel stability, raising the possibility of utilising 2OG to prevent, or at least slow the rate of, vascular decline in individuals with diabetes.

These novel findings expand our understanding of the metabolic and behavioural dysfunction that occurs in the MPs of people with diabetes. Furthermore, these data support the concept of using DM-2OG as a means of improving pericyte redox balance and mitochondrial function, while simultaneously allowing for enhanced pericyte–endothelial interaction. Given the relatively low sample size assessed in this study, if these effects are shown to be representative of people with diabetes then the potential of DM-2OG to slow the rate of vascular decline in vivo warrants further investigation.

## Electronic supplementary material

ESM (PDF 940 kb)

## Data Availability

The datasets generated during and/or analysed during the current study are available from the corresponding author on reasonable request.

## Abbreviations

CLI: Critical limb ischaemia
D-MP: Muscle pericyte from diabetic participant
DM-2OG: Dimethyl-2-oxoglutarate
EC: Endothelial cell
ECAR: Extracellular acidification rate
EGM2: Endothelial cell growth medium 2
GSH: Glutathione
GSSG: Glutathione (oxidised)
HIF1α: Hypoxia-inducible factor 1 α
HO-1: Haemoxygenase-1
IC-MS/MS: Anion-exchange chromatography–mass-spectrometry
LDHA: Lactate dehydrogenase A
MEM: Minimum essential media
MP: Muscle pericyte
MSC: Mesenchymal stromal cell
MyHC: Myosin heavy chain
ND-MP: Muscle pericyte from non-diabetic participant
NHDF: Adult normal human dermal fibroblast
OCR: Oxygen consumption rate
8-OHdG: 8-Hydroxy-2′-deoxyguanosine
2OG: 2-Oxoglutarate
PECAM: Platelet endothelial cell adhesion molecule
PFA: Paraformaldehyde
PKCβII: Protein kinase C, isoform β II
ROS: Reactive oxygen species
RT-qPCR: Quantitative RT-PCR
TCA: Tricarboxylic acid
