# Supplementary material for: Dimethyl-2-oxoglutarate improves redox balance and mitochondrial function in muscle pericytes of individuals with diabetes mellitus
Source: Diabetologia. 2020 Jul 30;63(10):2205–17. doi: 10.1007/s00125-020-05230-4 (PMC7476972; doi:10.1007/s00125-020-05230-4)
Supplement: Supplementary file 1 — (PDF 940 kb) [file 125_2020_5230_MOESM1_ESM.pdf]

# Dimethyl-2-oxoglutarate improves redox balance and mitochondrial function in muscle pericytes of individuals with diabetes mellitus

Ashton Faulkner[1,2*], Anita Tamiato[1], William Cathery[1], Andrea Rampin[3], Carlo Maria Caravaggi[3], Eva Jover[1], Steve Allen[4], Harry Mellor[2], David Hauton[5] Lisa Heather[6], Gaia Spinetti[3] and Paolo Madeddu[1*]

1. Bristol Medical School, Translational Health Sciences, University of Bristol, Upper Maudlin Street, Bristol BS2 8HW, UK
2. School of Biochemistry, University of Bristol, University Walk, Bristol, BS8 1TD, UK
3. IRCCS, MultiMedica, Milan, Italy
4. Department of Comparative Biomedical Sciences, Royal Veterinary College, London, UK
5. Department of Chemistry, University of Oxford, Oxford, UK
6. Department of Physiology, Anatomy & Genetics, University of Oxford, Oxford, UK

## Electronic Supplementary Material

## ESM methods

### Muscle pericyte isolation

Muscle biopsy samples were finely minced and digested with collagenase II (100 units/mL) for 45 min at 37°C with shaking. The digestion mixture was centrifuged and re-suspended in growth medium (α-minimum essential medium (α-MEM) supplemented with 20 % (vol/vol) foetal bovine serum (FBS) and 1 % (vol/vol) penicillin/streptomycin). The cell suspension was filtered through a 70-mm cell strainer, dispensed in plastic dishes at clonal density (1,000 cell/$cm^2$), and incubated in growth medium at 37 °C with 5 % $CO_2$. MPs were selected by plastic adherence in culture for at least 10 days when they form colonies positive for ALP, neural/glial antigen 2 (NG2), and CD146.

### Cell viability

MP viability was assessed using the Promega LDH-glo Viability assay following the manufacturer's instructions. α-MEM (10 % vol/vol FBS) medium samples were collected from 24 h cell cultures and diluted 1:50 in storage buffer (200 mmol/l Tris-HCl pH 7.3; 10 % vol/vol glycerol; 1 % wt/vol BSA). Samples were then mixed and incubated (60 min; room temperature) with kit-supplied LDH detection reagent and luminescence measured using a Promega Glo-Max Discover plate reader. Values were corrected for background and normalised to sample protein content.

### Proliferation

MP proliferation was quantified using a BrdU incorporation kit (Sigma Aldrich, UK) following the manufacturer's instructions. MPs were seeded in 96-well plates (1000 cells/well in triplicate) in the absence or presence of DM-2OG (1 mmol/l). Cells were cultured for 72 h, with medium and treatment refreshed every 24h. BrdU (10 µmol/l) was added for the final 24 h, after which, cells were fixed, and DNA denatured by addition of kit-supplied FixDenat solution (200 µl/well). Cells were then incubated with peroxidase-conjugated anti-BrdU-POD antibody (100 µl/well; 90 min) followed by substrate solution (100 µl/well; 30 min). Absorbance (405 nm) was measured using a Dinex-OpsysMR microplate reader.

### Permeability

HUVEC were seeded on transwell membrane inserts (100,000 cells/membrane) in EGM2 and allowed to attach for 24 h before the addition of MPs (25,000 cells/well) in EGM2. After 24 h, cells were incubated in EGM2 for a further 16 h in the absence or presence of 1 mmol/l DM-2OG, at which point medium was replaced with phenol red-free EGM2, either alone (bottom chamber) or supplemented with 0.5 mg/ml FITC-dextran (4 kDa or 70 kDa) (top chamber).

Permeability was assessed by measuring the accumulation of fluorescence within the bottom chamber using a Promega Glo-Max Discover plate reader.

**Anion-exchange chromatography–mass-spectrometry (IC-MS/MS)**

Following 16 h incubation with or without 1 mmol/l DM-2OG, medium was removed, and cells rapidly washed in PBS before the addition of ice-cold 80% vol/vol methanol (500 µl/10 cm dish). Plates were incubated on ice for 3 min before material was collected, centrifuged (13,000 rpm; 30 min), and filtered using a 10 kD molecular weight cut-off filter. A quality control (QC) sample was made through combining equal volumes of each sample (250 µl final volume) to monitor analytical reproducibility. IC-MS/MS was performed using an ICS-5000+ HPLC system coupled directly to a Q-Exactive HF Hybrid Quadrupole-Orbitrap mass spectrometer with a HESI II electrospray ionisation source (Thermo Scientific, San Jose, CA). The ICS-5000+ HPLC system incorporated an electrolytic anion generator (KOH) which was programmed to produce a $OH^-$ gradient over 37 min. An inline electrolytic suppressor removed $OH^-$ ions and cations from the post-column eluent stream prior to MS analysis (ThermoScientific Dionex AERS 500). A 10 µl partial-loop injection was used for all analyses and the chromatographic separation was performed using a ThermoScientific Dionex IonPac AS11-HC 2×250 mm, 4 µm particle size column with a Dionex Ionpac AG11-HC 4 µm 2x50 guard column inline. The IC flow rate was 0.250 ml/min. The total run time was 37 min and the hydroxide ion-gradient comprised as follows: 0 min, 0 mmol/l; 1 min, 0 mmol/l; 15 min, 60 mmol/l; 25 min, 100 mmol/l; 30 min, 100 mmol/l; 30.1 min, 0 mmol/l; 37 min, 0 mmol/l. Analysis was performed in negative-ion mode using a scan-range from m/z 60-900 and resolution set to 70,000. The tune file source parameters were set as follows: Sheath gas flow 60 ml/min; Aux gas flow 20 ml/min; Spray voltage 3.6 v; Capillary temperature 320 °C; S-lens RF value 70; Heater temperature 350 °C. AGC target was set to 1e6 v ions and the Max IT value was 250 ms. The column temperature was kept at 30 °C throughout the experiment. Full scan data were acquired in continuum mode. Raw data files were processed using ProgenesisQI (Waters, Elstree, UK) and compounds were identified with reference to accurate mass and retention time compared with authenticated standards run in-house. All data was normalised to total DNA for each sample and expressed as relative abundance. Absolute abundance of adenine nucleotides (ATP/AMP/ADP) was calculated with reference to standard curves.

**Seahorse extracellular flux analysis**

Cells (20,000 cells/well) were seeded into XFp tissue culture plates in the absence or presence of DM-2OG (1 mmol/l). Following 16 h incubation (37 ˚C, 5 % $CO_2$), cells were washed (x 3) with assay basal medium supplemented with sodium pyruvate (1 mmol/l), L-glutamine (2 mmol/l) and D-glucose (5 mmol/l), before an additional 1 h incubation in assay medium in the

absence of $CO_2$. Plates were then transferred to the XFp machine, baseline readings were obtained, followed by the sequential addition of oligomycin (2 μmol/l), FCCP (2 μmol/l) and rotenone/antimycin-A (0.5 μmol/l each). Three readings (5 min intervals) were obtained after each pharmacological agent and values normalised to protein content.

**Extracellular lactate accumulation**

MPs were incubated (24 h) in the absence or presence of 1 mmol/l DM-2OG under normoxia (21 % $O_2$) or mild hypoxia (2 % $O_2$). Medium was collected, centrifuged (10,000 g; 5 min), and stored at -80 °C until analysed. Medium lactate content was measured using ABX Pentra C200 chemistry analyser with background-corrected (medium alone) results normalised to protein content.

**Measurement of $H_2O_2$**

Medium $H_2O_2$ level was measured using the Promega ROS-Glo™ $H_2O_2$ Assay following the manufacturer's instructions. MPs (1000 cells/well 96-well plate) were incubated in the presence or absence of DM-2OG (1 mmol/l) for 16 h (in triplicate). Kit-supplied $H_2O_2$ Substrate Solution (25 μmol/l) was added for the final 6 h of treatment, followed by the addition of Kit-supplied ROS-Glo Detection Solution. Plates were incubated for 20 min at room temperature before luminescent signal was measured using a Promega Glo-Max Discover luminometer.

**Measurement of GSH/GSSG**

GSH was measured using the Promega GSH/GSSG-Glo Assay kit following the manufacturer's instructions. Cells were incubated for 16 h in the absence or presence of 1 mmol/l DM-2OG. An equal volume of either Total Glutathione Lysis reagent or Glutathione Oxidized Lysis reagent (kit-supplied) was added, and the plate incubated for 5 min with orbital shaking. Kit-supplied Luciferin Generation reagent was added to all wells (30 min), followed by an equal volume of kit-supplied Luciferin Detection reagent (15 min). Luminescent signal was measured using a Promega Glo-Max Discover luminometer with results normalised to protein content. GSH/GSSG ratio was calculated as GSH-GSSG/(GSSG/2).

## ESM Tables

### ESM Table 1: Characteristics of the study population

| | Non-diabetic control patients n = 5 | Patients with Diabetes mellitus n = 5 | *p*-value (*t*-test) |
|---|---|---|---|
| Age (years) (mean ± SEM) | 48.8 ± 4.95 | 58.8 ± 5.11 | 0.161 |
| Sex | Male: 4 Female: 1 | Male: 3 Female: 2 | - |
| Type of diabetes | - | Type-1: 2 Type-2: 3 | - |
| Fasting glucose (mmol/l) (mean ± SEM) | 5.22 ± 0.14 | 6.42 ± 0.71 | 0.134 |
| HbA1c (mmol/mol) (mean ± SEM) | - | 65.60 ± 7.66 | - |
| HbA1c (%) (mean ± SEM) | - | 8.16 ± 0.70 | - |
| Critical limb ischaemia | No | Yes | - |

## ESM Table 2: Primer sequences used for RT-qPCR

| Target gene | Primer Sequence | | NCBI Accession number / Reference |
|---|---|---|---|
| *GLUT1* | Forward | TGGCATCAACGCTGTCTTCT | NM_006516.2 |
| | Reverse | CTAGCGCGATGGTCATGAGT | |
| *GLUT4* | Forward | TAGGCTCCGAAGATGGGGAA | NM_001042.2 |
| | Reverse | GGAAAAGATGGCCACGGAGA | |
| *LDHA* | Forward | TTGAAGGGAGAGATGATGGA | NM_005566 |
| | Reverse | CCAGCCGTGATAATGACCAG | |
| *CAT* | Forward | TCTCACCAAGGTTTGGCCTC | NM_001752.3 |
| | Reverse | CGGTGAGTGTCAGGATAGGC | |
| *HMOX-1* | Forward | CTTCTTCACCTTCCCCAACA | [1] |
| | Reverse | TTCTATCACCCTCTGCCTGA | |
| *GCLC* | Forward | TCCAGGTGACATTCCAAGCC | NM_001498.4 |
| | Reverse | GAAATCACTCCCCAGCGACA | |
| *TBP* | Forward | GGAGAGTTCTGGGATTGTAC | [2] |
| | Reverse | CTTATCCTCATGATTACCGCAG | |

**ESM Table 3: Antibodies used in western blotting**

| Antibody | Vendor | Catalogue number | Dilution |
|---|---|---|---|
| anti-β-actin | Sigma Aldrich; UK | A5441 | 1:10,000 |
| anti-PKCβII | Abcam; UK | ab32026 | 1:500 |
| anti-SHC | Abcam; UK | ab24787 | 1:1000 |
| anti-HO1 | Abcam; UK | ab13243 | 1:1000 |
| anti-GLUT1 | Abcam; UK | ab115730 | 1:5000 |
| anti-Catalase | Cell Signaling Technologies; UK | D4P7B | 1:1000 |
| anti-LDHA | Cell Signaling Technologies; UK | 2012 | 1:1000 |

**ESM Table 4: Antibodies used in immunocytochemistry**

| Antibody | Vendor | Catalogue number | Dilution |
|---|---|---|---|
| anti-PDGFRβ | R&D Systems, UK | AF385 | 1:50 |
| anti-CD31 | R&D Systems, UK | BBA7 | 1:50 |
| anti-CD146 | Abcam; UK | ab75769 | 1:100 |
| anti-NG2 | Merk Millipore; UK | AB5320 | 1:100 |

## ESM figures

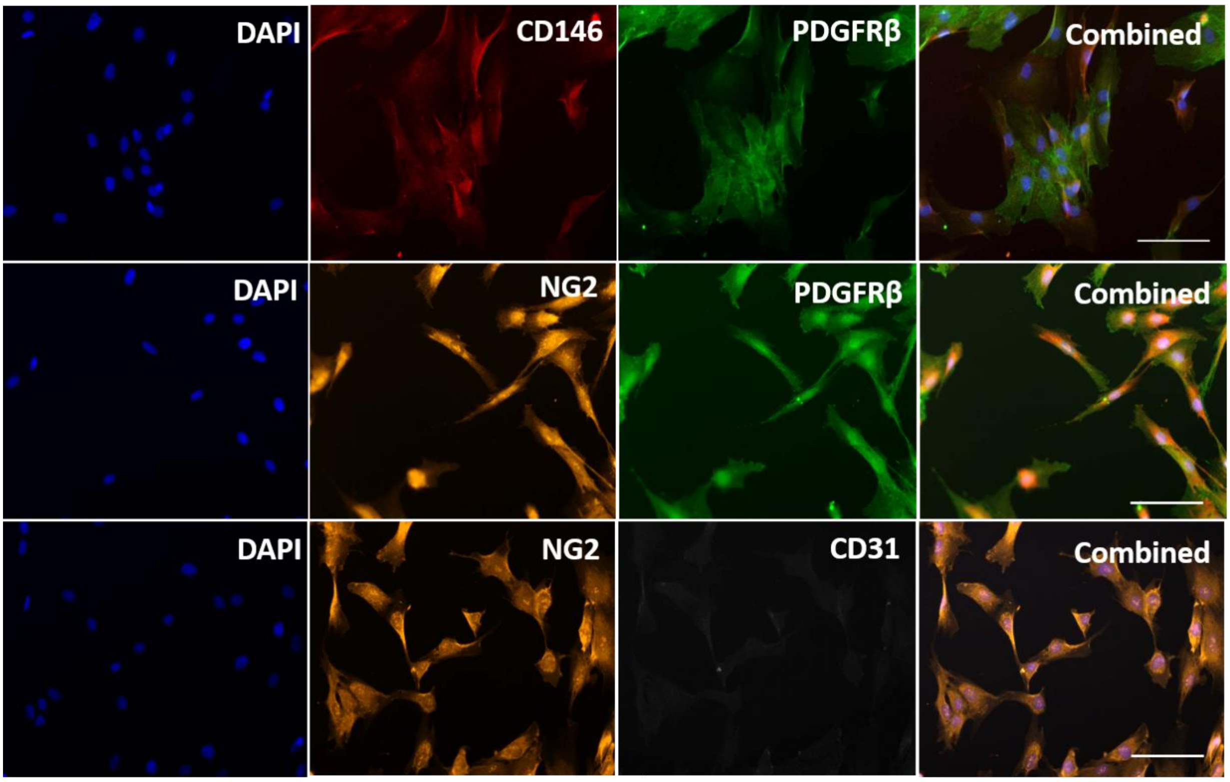


**ESM fig 1:** Immunofluorescence images (20x objective; scale bar = 50µm) showing that isolated pericyte-like cells (muscle pericytes; MPs) are positive for the typical pericyte markers CD146 (red), PDGFRβ (green) and NG2 (orange), and negative for the endothelial marker, CD31 (white). Each row represents an individual well.

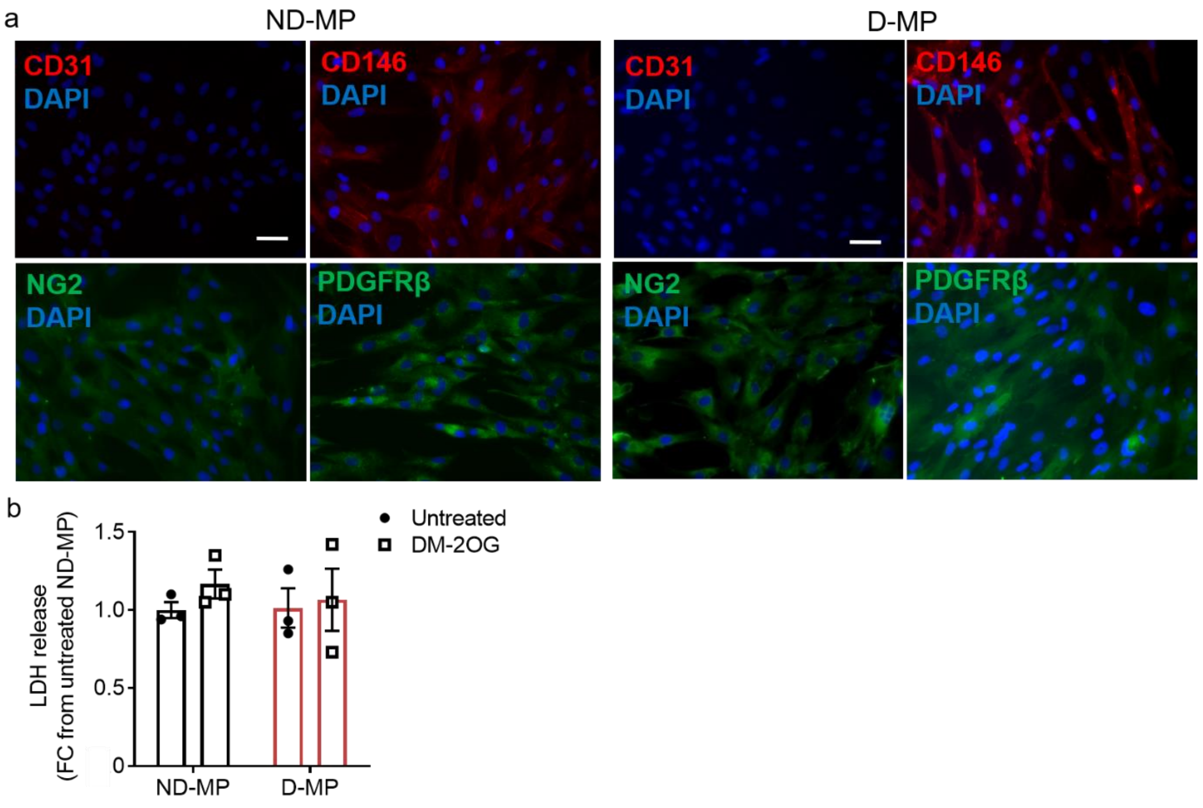


**ESM fig 2: (a)** Representative immunofluorescence images (scale bar = 50 µm) showing that ND- and D-MPs express similar levels of CD146, PDGFRβ and NG2, and are negative for CD31. **(b)** Viability of isolated cells, as assessed using the LDH release assay (LDH-Glo assay; Promega, UK), is not significantly different between ND- and D-MPs and is not affected by supplementation with DM-2OG.

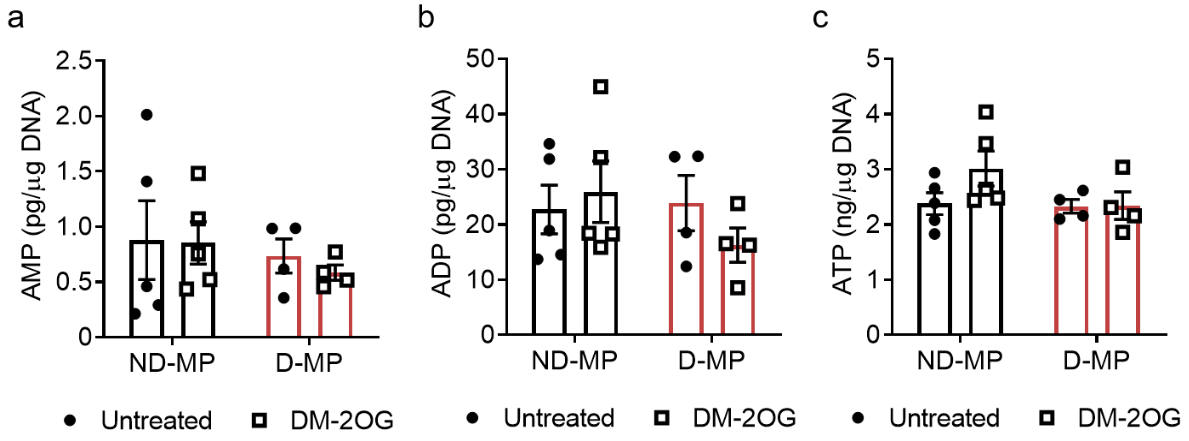


**ESM fig 3:** AMP **(a)**, ADP **(b)** and ATP **(c)** adenine nucleotide abundance is not significantly different between ND- and D-MPs or following supplementation with DM-2OG (1 mmol/l; 16h).

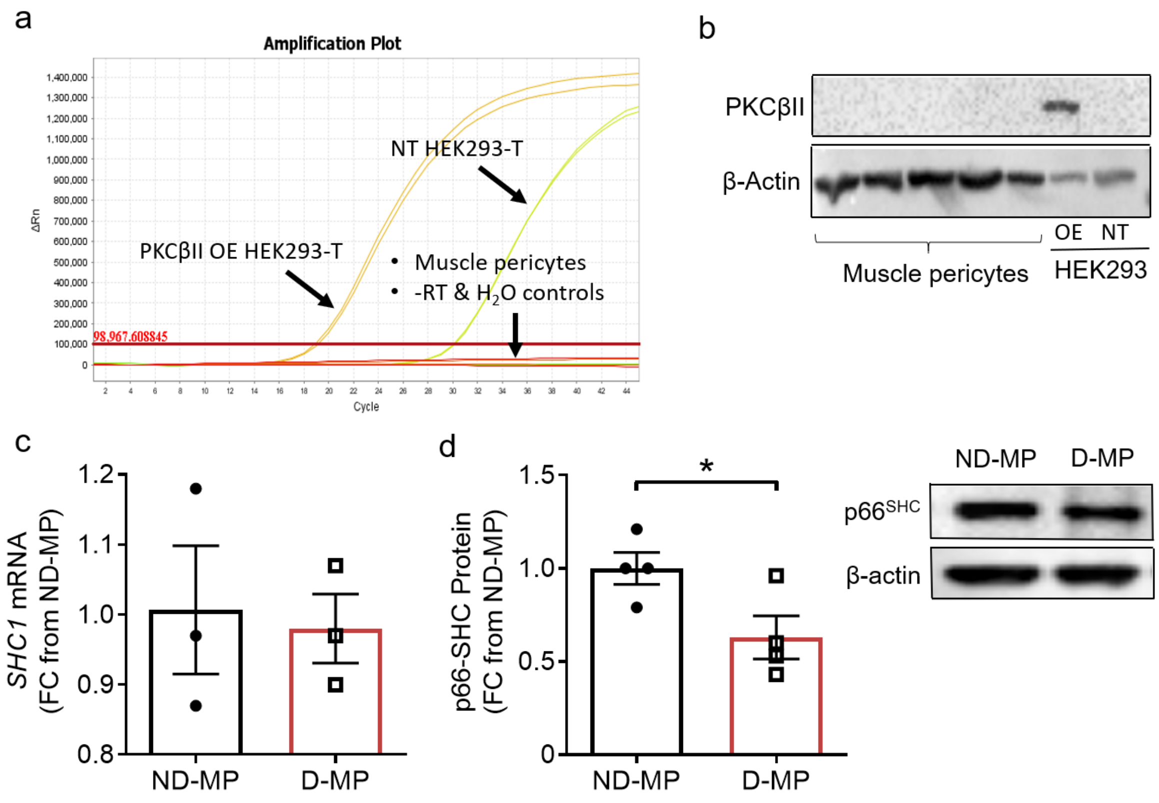


**ESM fig 4:** Amplification plot from RT-qPCR analysis **(a)** and western blot **(b)** showing that PKCβII is not detected in either ND- or D-MPs, with PKCβII-overexpressing HEK293-T cells acting as a positive control. **(c)** *SHC1* mRNA expression is not significantly different between ND- and D-MPs. **(d)** Densitometry analysis and representative western blot showing that $p66^{SHC}$ is significantly reduced in D-MPs compared with ND-MPs. Data represent means (± S.E.M) from $n$ = 3. * $p$<0.05 as determined by Student's $t$-test. See ESM fig. 7 for original unedited blot.

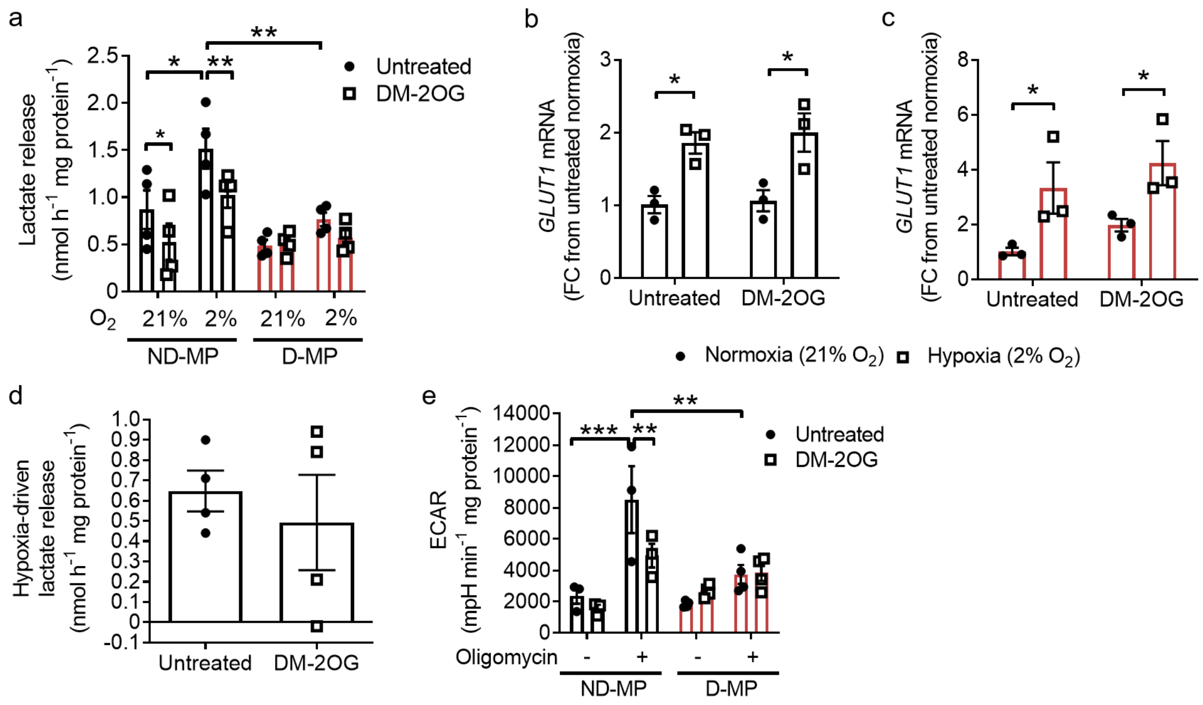


**ESM fig 5: (a)** Lactate accumulation within the culture medium is lower in D-MPs compared with ND-MPs under both normoxia (21 % $O_2$) and mild hypoxia (2 % $O_2$). Supplementation with 1 mmol/l DM-2OG reduces lactate release in ND-MPs but has no effect on D-MPs. Data are means (± S.E.M) of $n$ = 4; * $p$<0.05 ** $p$<0.01 as determined by two-way ANOVA followed by Bonferroni's post-comparison test. *GLUT1* mRNA is significantly increased under mild hypoxia (2%) in ND-MPs (**b**) and D-MPs (**c**) in the absence and presence of DM-2OG. Data are mean (± S.E.M) of $n$ = 3; * $p$<0.05 as determined by two-way ANOVA followed by Bonferroni's post-comparison test. **(d)** The change in medium lactate accumulation in response to mild hypoxia (2 % $O_2$) is not significantly different between untreated and DM-2OG-treated ND-MPs. **(e)** Extracellular acidification rate (ECAR) is lower in untreated D-MPs compared with ND-MPs and following treatment with oligomycin (2 µmol/l). Supplementation with DM-2OG reduces ECAR in ND-MPs but has no effect on D-MPs. Data are means (± S.E.M) of $n$ = 3 for ND-MP and $n$ = 4 for D-MP; ** $p$<0.01 *** $p$<0.001 as determined by two-way ANOVA followed by Bonferroni's post-comparison test.

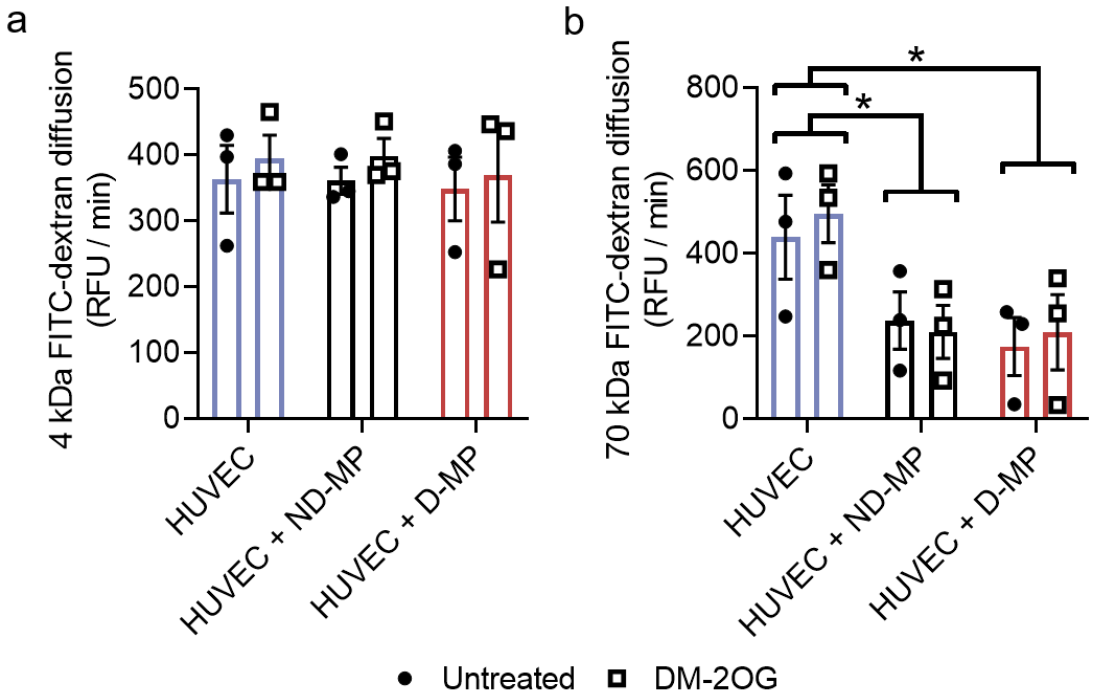


**ESM fig 6: (a)** Neither the presence of MPs nor DM-2OG (1 mmol/l) has an effect on HUVEC permeability of small molecular weight FITC-dextran (4 kDa) **(b)** ND- and D-MPs significantly reduce the permeability of HUVEC monolayers to large molecular weight FITC-dextran (70 kDa), with no effect of DM-2OG supplementation. Data are mean (± SEM) of $n = 3$; * $p<0.05$ as determined by two-way ANOVA followed by Bonferroni's post-comparison test.

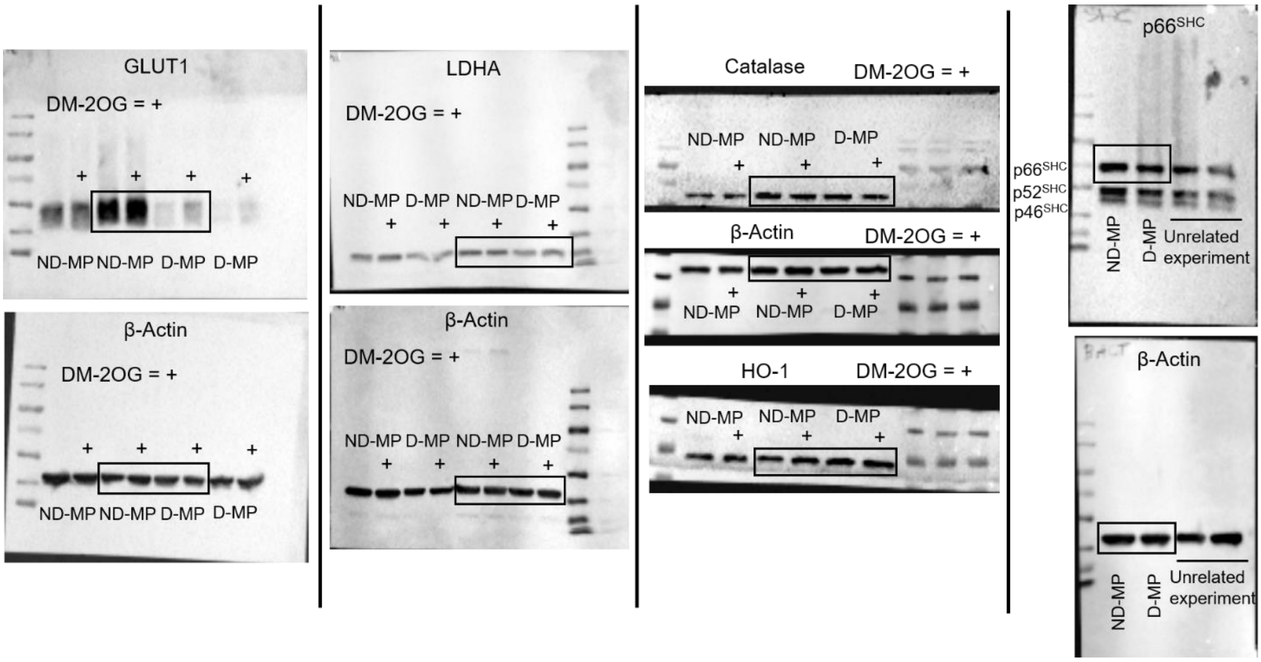


**ESM fig 7:** Original unedited western blots used for figures 3d and 6c, and ESM fig. 4d. Boxed areas indicate bands used for final figures.

## Supplementary references

[1] Ali F, Ali NS, Bauer A, et al. (2010) PPARδ and PGC1α act cooperatively to induce haem oxygenase-1 and enhance vascular endothelial cell resistance to stress. Cardiovascular research 85(4): 701-710

[2] Goldberg MS, Sharp PA (2012) Pyruvate kinase M2-specific siRNA induces apoptosis and tumor regression. The Journal of Experimental Medicine 209(2): 217-224
